# A Novel Surrogate Nomogram Capable of Predicting OncotypeDX Recurrence Score©

**DOI:** 10.3390/jpm12071117

**Published:** 2022-07-08

**Authors:** Matthew G. Davey, Amirhossein Jalali, Éanna J. Ryan, Ray P. McLaughlin, Karl J. Sweeney, Michael K. Barry, Carmel M. Malone, Maccon M. Keane, Aoife J. Lowery, Nicola Miller, Michael J. Kerin

**Affiliations:** 1 The Lambe Institute for Translational Research, National University of Ireland, H91 TK33 Galway, Ireland; aoife.lowery@nuigalway.ie (A.J.L.); nicola.miller@nuigalway.ie (N.M.); michael.kerin@nuigalway.ie (M.J.K.); 2 Department of Surgery, Galway University Hospitals, H91 YR71 Galway, Ireland; eannaryan@rcsi.ie (É.J.R.); ray.mclaughlin@hse.ie (R.P.M.); karlj.sweeney@hse.ie (K.J.S.); kbarsurg@gmail.com (M.K.B.); carmel.malone@nuigalway.ie (C.M.M.); 3 Department of Mathematics and Statistics, University of Limerick, V94 T9PX Limerick, Ireland; amir.jalali@ul.ie; 4 School of Medicine, University of Limerick, V94 T9PX Limerick, Ireland; 5 Department of Medical Oncology, Galway University Hospitals, H91 YR71 Galway, Ireland; maccon.keane@nuigalway.ie

**Keywords:** breast cancer, genomics, personalized medicine, surgical oncology

## Abstract

Background: OncotypeDX Recurrence Score© (RS) is a commercially available 21-gene expression assay which estimates prognosis and guides chemoendocrine prescription in early-stage estrogen-receptor positive, human epidermal growth factor receptor-2-negative (ER+/HER2−) breast cancer. Limitations of RS testing include the cost and turnaround time of several weeks. Aim: Our aim is to develop a user-friendly surrogate nomogram capable of predicting RS. Methods: Multivariable linear regression analyses were performed to determine predictors of RS and RS > 25. Receiver operating characteristic analysis produced an area under the curve (AUC) for each model, with training and test sets were composed of 70.3% (*n* = 315) and 29.7% (*n* = 133). A dynamic, user-friendly nomogram was built to predict RS using R (version 4.0.3). Results: 448 consecutive patients who underwent RS testing were included (median age: 58 years). Using multivariable regression analyses, postmenopausal status (β-Coefficient: 0.25, 95% confidence intervals (CIs): 0.03–0.48, *p* = 0.028), grade 3 disease (β-Coefficient: 0.28, 95% CIs: 0.03–0.52, *p* = 0.026), and estrogen receptor (ER) score (β-Coefficient: −0.14, 95% CIs: −0.22–−0.06, *p* = 0.001) all independently predicted RS, with AUC of 0.719. Using multivariable regression analyses, grade 3 disease (odds ratio (OR): 5.67, 95% CIs: 1.32–40.00, *p* = 0.037), decreased ER score (OR: 1.33, 95% CIs: 1.02–1.66, *p* = 0.050) and decreased progesterone receptor score (OR: 1.16, 95% CIs: 1.06–1.25, *p* = 0.002) all independently predicted RS > 25, with AUC of 0.740 for the static and dynamic online nomogram model. Conclusions: This study designed and validated an online user-friendly nomogram from routinely available clinicopathological parameters capable of predicting outcomes of the 21-gene RS expression assay.

## 1. Introduction

OncotypeDX Recurrence Score© (RS) (Genomic Health Inc., Redwood City, CA, USA) is a commercially available, clinically validated 21-gene expression assay which predicts the risk of distant disease recurrence in early-stage estrogen receptor-positive, human epidermal growth factor receptor-2-negative (ER+/HER2−) breast cancer [1,2,3]. The 21-gene expression assay successfully substratifies patients who will derive the most benefit from combined chemoendocrine therapy prescription, leading to the appropriate de-escalation of chemotherapy prescription for a large proportion of these patients [4]. Accordingly, RS testing has facilitated the personalization of cancer treatment for those with early-stage ER+/HER2− disease, leading to the endorsement of the 21-gene expression assay by several expert panels and oncology societies in their recommended guidelines for early-stage breast cancer, including the American Society for Clinical Oncology (ASCO), the National Comprehensive Cancer Network (NCCN), and the European Society of Medical Oncology (ESMO), among others [5,6,7,8,9]. 

There are several drawbacks to using RS testing in modern clinical practice: Analysis of tumor specimens using the 21-gene expression assay is only conducted in the Genomic Health Inc. headquarters in California, which incurs a local turnaround time of several weeks in the Republic of Ireland. Although the initial aspiration for the assay was to provide a cost-effective, personalized approach to chemoendocrine prescription in ER+/HER2− early-stage breast cancer [10,11], novel data has emerged challenging this original perception, suggesting the true cost-effectiveness of the assay in clinical practice [12,13]. Moreover, the local cost of each RS test is approximately €3000 [14]. Additionally, although RS testing is publicly funded in the Republic of Ireland [14], this assay is not publicly funded in many countries worldwide, which exacerbates the ongoing global healthcare inequity [15]. Orucevic et al. previously outlined that RS testing is performed on just one-third of potentially eligible patients in the United States [16], while Albanell et al. reported that just 20% of eligible European citizens undergo RS testing [17]. Furthermore, the availability of RS testing has been illustrated to be directly influenced by ethnic race and socioeconomic class [18,19,20]. Therefore, there remains an unmet need to develop a surrogate biomarker which rivals RS to aid therapeutic decisions making for those with early-stage ER+/HER2− disease. 

It is well-described that a large component of RS testing can be determined through the procurement and assessment of routine clinicopathological and immunohistochemical (IHC) tumor characteristics [21,22,23]. Previous work from our group demonstrated that certain clinicopathological parameters (such as steroid hormone receptor status and tumor grade) can successfully predict RS [24], to guide and facilitate cost-effective therapeutic decision making in poorly resourced healthcare economies, who have limited access to multigene expression assays, such as RS. Similarly, the aim of this study was to develop a user-friendly nomogram which could be used as a surrogate prediction model capable of predicting the results of the 21-gene RS expression assay. The nomogram development is based on consecutive patients diagnosed and treated with curative intent in a large, tertiary referral center in the Republic of Ireland, using seven readily available clinicopathological variables which correlate with RS in early-stage breast cancer.

## 2. Methods

Local hospital ethical approval was granted from the Galway University Hospitals Clinic Research Ethics Committee (C.A.2377). Patient care was provided in a large tertiary referral center providing services supporting breast cancer diagnosis and treatment at Galway University Hospital (GUH) in the Republic of Ireland. GUH has Organisation of European Cancer Institute (OECI) accreditation for the provision of cancer care to the population living in the west of Ireland.

### 2.1. Study Design

A single-center, retrospective cohort study was undertaken, which included consecutive patients diagnosed with ER+/HER2− early-stage breast cancer who underwent RS testing on their resected tumor specimen during a 9-year period (January 2007–December 2015). Patients who had metastatic disease in the axillary lymph nodes were excluded, as were patients presenting with metastatic disease at presentation (M1). Detailed information with respect to demographics, clinicopathological data, and RS testing results were collected using electronic patient records. Included patients were identified from a prospectively maintained database at the Department of Surgery at the National University of Ireland, Galway.

### 2.2. Diagnosis and Staging

Included patients underwent comprehensive triple assessment to facilitate accurate diagnosis in our tertiary referral center. This involved the following: (1) clinical breast examination performed by a consultant breast surgeon; (2) radiological assessment performed via mammography and/or ultrasound, with magnetic resonance imaging used in select cases. All imaging was then analyzed by a consultant radiologist with specialist training in breast pathology; and (3) diagnostic core biopsies were performed under image guidance by the consultant radiologist, before diagnosis was confirmed via histological analysis of tumor specimens using a standardized reporting template. Specimens were analyzed in an accredited pathology laboratory, with formal validation by a specialist consultant breast pathologist. Staging was performed in accordance with the American Joint Committee on Cancer (AJCC), version 8 Guidelines [25].

### 2.3. Histopathology Tumor Assessment

Evaluation of estrogen (ER) and progesterone receptor (PgR) statuses was performed in accordance with the Allred scoring system [26]. Assessment of human epidermal growth factor receptor-2 (HER2) status was determined using IHC, with tumors scoring 2+ then submitted for fluorescence in-situ hybridization (FISH) to confirm HER2 tumor status [27,28,29]. Tumor grading was evaluated using the Elston Ellis modification of the Scarff–Bloom–Richardson grading system (in accordance with the World Health Organization Classification of Tumors Guidelines) [30,31]. RS testing was performed through reverse-transcription polymerase chain reaction at the Genomic Health laboratory (Redwood City, CA, USA), using paraffin-embedded tumor tissue samples, as previously outlined in the seminal work of Paik et al. [1]. RS is a composite score of 16 cancer-related and 5 reference genes which are incorporated into an algorithm which generates a recurrence score of 0–100, which represents the patient’s individual risk of distant disease recurrence at 9 years following treatment.

### 2.4. Statistical Analysis

Descriptive statistics were used to record clinicopathological and RS data from 448 patients who underwent RS testing for ER+/HER2− breast cancers in our center. The dataset is randomly divided into a training set, which is used to train a model for predicting the RS, and an independent test set to evaluate the model performance. The training set comprised 70.3% of the total cohort (*n* = 315) and the test set comprised 29.7% (*n* = 133). Multivariable linear regression analyses were performed to determine predictors of RS, with results expressed as β-Coefficient with associated 95% confidence intervals (CIs). *p*-values with *p* < 0.050 were determined to be statistically significant. Various graphical and numerical performance outcome summaries were used to demonstrate the discrimination ability of the model. Receiver operating characteristic (ROC) curves were used to demonstrate graphically how best the model predict would predict RS [32]. ROC analysis produces an area under the curve (AUC) for each model by plotting the sensitivity and specificity of the model at each of its risk thresholds. The AUC value, in addition to the accuracy, sensitivity, specificity, positive predictive value (PPV), and negative predictive value (NPV), are utilized to evaluate the model performance numerically [33]. For the initial analysis, RS was expressed as a continuous variable, however, it is also classified as ≤25 or >25 in accordance with the clinically relevant cut-offs established by Sparano et al. in the TAILORx [2]. Finally, a dynamic nomogram (https://mattdavey93.shinyapps.io/RSsurrogate/) (accessed on 13 December 2021) [34] was designed and built to predict RS with the relevant uncertainty for any given user information [35]. This web application aims to convey the result of the RS surrogate risk calculator in the most translated way for oncologists faced with decisions with respect to chemoendocrine therapy prescription in the absence of RS. Data analysis is performed in R (version 4.0.3) using ‘plyr’, ‘caret’, ‘car’, ‘boot’, ‘pROC’, ‘rms,’ and ‘DynNom’ packages.

## 3. Results

### 3.1. Clinicopathological Dataset

In total, 448 consecutive patients who underwent RS testing for ER+/HER2− breast cancer were included. The median age at diagnosis was 58 years (interquartile range (IQR): 51–64 years). Overall, 69.0% of patients were postmenopausal at diagnosis (311/448) and 65.0% presented via the symptomatic referral pathway (289/448). The median tumor size was 20.0 mm (IQR: 14.0–26.0 mm). In total, 97.5% of tumors were T-stage 1–2 (437/448), 76.1% had invasive ductal carcinoma (IDC) histology (341/448), and 70.5% had grade 2 disease (296/420). All patients had ER+ (100.0%, 448/448) and 86.1% had progesterone receptor-positive (PgR+) disease (386/448). The median RS was 17 (IQR: 13–22, range: 0–44). All patients had node-negative disease in the axilla (100.0%, 448/448). Summary data for the matched training cohort (70.3%, *n* = 315) and test cohort (29.7%, *n* = 133) are outlined in Table 1. 

### 3.2. Nomogram Model Development

First, we attempted to create a surrogate nomogram model capable of predicting RS as a continuous variable using our training dataset (*n* = 315, 70.3%). Using multivariable linear regression analyses, being premenopausal (β-Coefficient: 0.25, 95% CIs: 0.03–0.48, *p* = 0.028), having grade 3 disease (β-Coefficient: 0.28, 95% CIs: 0.03–0.52, *p* = 0.026), and ER score (β-Coefficient: −0.14, 95% CIs: −0.22– −0.06, *p* = 0.001) all independently predicted RS (Table 2). 

**Figure 1:**
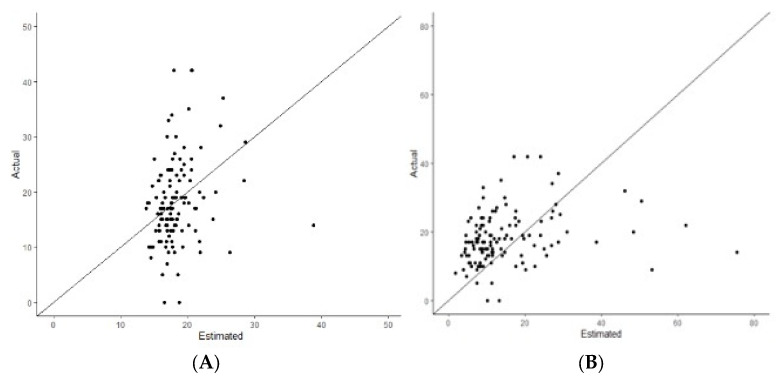
Calibration plots for the nomogram models illustrating the predicted probability for (**A**) recurrence score as a continuous outcome, and (**B**) as a binary outcome for recurrence score ≤ 25 and >25.

Using our training dataset, we created a nomogram model capable of predicting high-risk RS (RS > 25). Using multivariable logistic regression analyses, having grade 3 disease (odds ratio (OR): 5.67, 95% CIs: 1.32–40.00, *p* = 0.037), decreased ER score (OR: 1.33, 95% CIs: 1.02–1.66, *p* = 0.050), and decreased progesterone receptor score (OR: 1.16, 95% CIs: 1.06–1.25, *p* = 0.002) all independently predicted RS > 25 (Table 3). The calibration plot for these models illustrating the predicted probability for RS versus the observed RS are illustrated in Figure 1. 

### 3.3. Performance of Nomogram Models

**Figure 2:**
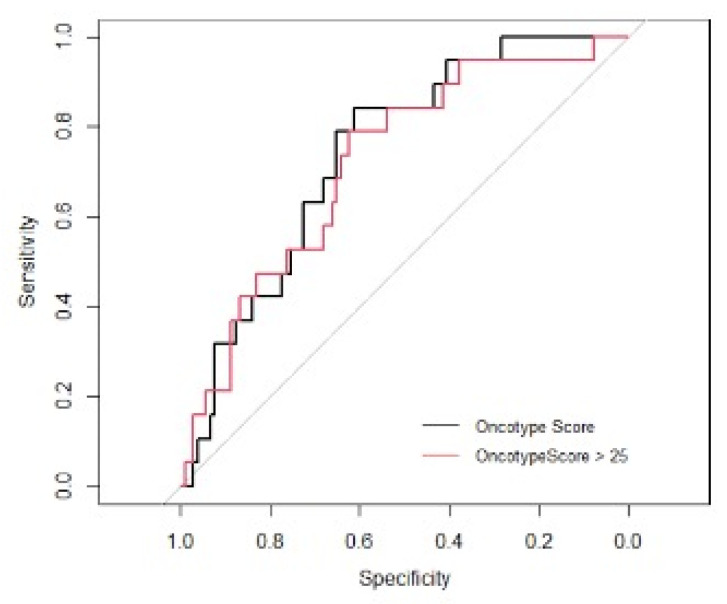
Receiver operating characteristic curve analyses performed to assess the diagnostic test accuracy of the nomogram models for recurrence score as a continuous variable (area under the curve: 0.719) and recurrence score ≤ 25 and >25 (area under the curve: 0.740).

**Figure 3:**
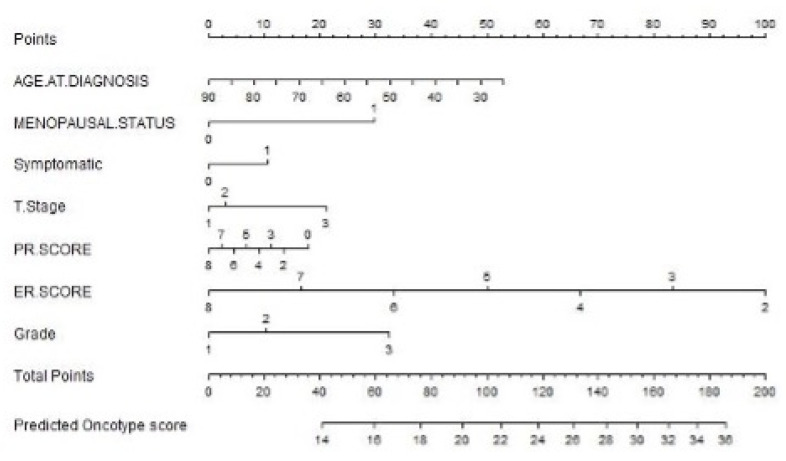
Static nomogram model capable of predicting a ‘surrogate’ recurrence score as a continuous variable.

ROC curve analyses were performed to test the diagnostic test accuracy of the nomogram models, with associated calibration plots for nomogram models for predicting RS as a continuous variable (Figure 1A) and as a binary outcome of RS ≤ 25 or RS > 25 (Figure 1B). Using our test (or validation) set (*n* = 133, 29.7%), the AUC for the nomogram model assessing RS was 0.719 (Figure 2). Overall, the accuracy of this nomogram was 0.800 (95% CI: 0.719–0.866) with a sensitivity of 0.872, specificity of 0.312, PPV of 0.887, and NPV of 0.316. Additionally, using our test set (or validation set), the AUC for the nomogram model assessing RS > 25 was 0.740 (Figure 2). Overall, the accuracy of the second nomogram was 0.858 (95% CI: 0.764–0.889) with a sensitivity of 0.858, specificity of 0.400, PPV of 0.972, and NPV of 0.105. This model was selected to develop the static (Figure 3) and dynamic (https://mattdavey93.shinyapps.io/RSsurrogate/) (accessed on 13 December 2021)) nomogram development. Due to the slightly enhanced diagnostic test accuracy of both nomogram models, the second surrogate nomogram (which predicted RS as a binary variable) was selected for use in the static and dynamic nomograms due to this model providing more information which is of use to the multidisciplinary team when making decisions about chemoendocrine prescription.

## 4. Discussion

Contemporary oncological practice now recognizes the idiosyncratic genetic genotypic and phenotypic profiles of each breast tumor, which has facilitated the substratification of the disease into biologically diverse subgroups, even within the same molecular subtypes [36,37]. Thus, the molecular era has facilitated the personalization of the management of early-stage ER+/HER2− breast cancer through patient and tumor differentiation by multigene panels, such as the 21-gene RS expression assay [1,2,38]. Unfortunately, as outlined previously, there are several disadvantages to these commercial profiling assays [10,11,12,14], which set the foundations for the conduction of the current study. We successfully designed and validated an online user-friendly nomogram using routinely available clinicopathological data that could accurately predict RS (accuracy: 0.840, 95% CI: 0.764–0.899). A dynamic version of this nomogram has been made readily available online, which may be advantageous in serving populations being treated with ER+/HER2− disease in inadequately resourced and underfunded healthcare systems across the globe. While several previous authors have established that RS can be predicted from readily available clinicopathological variables (i.e.,: ER, PgR, grade, menopause status, etc.) [21,22,23,24], the current study has built on this hypothesis through the incorporation of such parameters into multivariable models, which have facilitated nomogram development to aid RS estimation. Thus, this study contributes to the current efforts by clinicians to balance healthcare inequity across the world, as demonstrated in previous global surgery initiatives [39]. 

It is important to note that the current nomograms pragmatically use steroid hormone receptor and menopause status as key determinants of RS. Clarifying menopause status and measurement of ER/PgR is mandated for all cases of invasive breast cancer, as recently outlined in the 5th ESMO International Consensus for advanced and local breast cancers in addition to the recent RxPONDER trial [3,40,41]. Therefore, using these critical parameters to estimate RS is crucial for patients being treated in poorly resourced healthcare settings. This is not the first study to investigate building a nomogram to predict RS; from their series of 485 patients with ER+/HER2− breast cancer with 0–3 positive lymph nodes, Lee et al. developed a nomogram which demonstrated the ability of ER score, PgR score, tumor grade, lymphovascular invasion (LVI), and Ki-67 to predict low-risk RS (RS < 25) [42]. Of note, two of the five variables (LVI and Ki-67) used by these authors to predict RS were not included in our nomogram: LVI is not conducted as routine on every specimen in the setting of node negative ER+/HER2− cancers in our center due to the limited premise in best practice guidelines for using the biomarker to guide adjuvant therapeutic treatment decisions in early-stage breast cancer [40,43,44,45,46]. Similarly, Ki-67 measurement is not mandated due to inconsistencies in detection [47]. Therefore, reliance upon such biomarkers to provide a reproducible and equitable surrogate biomarker to RS may be brought into question. Although measurement is not mandated for all cases of invasive breast cancer, we must acknowledge that LVI is an early indicator of metastatic dissemination [48] and has recently been reported as a significant predictor of recurrence-free (hazard ratio (HR): 1.425), disease-free (DFS) (HR: 1.345), and overall survival (OS) (HR: 1.345) outcomes in an analysis of 17,322 patients by Houvenaeghel et al. [49]. Moreover, data from the aforementioned study suggests that LVI has potential to serve as a surrogate biomarker of RS predictive of patients with luminal A disease who may benefit from adjuvant chemotherapy prescription. Similarly, Lee et al. describe Ki-67 indices as predictive correlates of RS in their nomogram, with results validated by other authors in two more recent studies which developed surrogate nomograms to RS [50,51]. This is an unsurprising finding; within the 21-gene expression assay, comparison between the 16 cancer-related and 5 reference (or “housekeeping”) genes is included in an algorithm to generate the RS [1]. Of the 16 cancer genes in the panel, 5 are directly related to proliferation, with 1 representing and corresponding with Ki-67 antigen expression [14,47], making it foreseeable that several reports correlate RS and Ki-67 protein expression in ER+ disease [52,53,54]. Therefore, inclusion of Ki-67 expression indices is justified in previous nomograms. At present, Ki-67 proliferation indices are not routinely performed for every specimen with ER+/HER2− early breast cancer due to its limited value for treatment decisions due to inconsistent and questionable analytical validity and reproducibility [47], as recently outlined by the International Ki-67 in Breast Cancer working group [55]. Ovucevic et al. previously developed a surrogate prediction nomogram model from 27,719 patients using six clinicopathological variables (age at diagnosis, tumor size, tumor grade, PgR status, LVI, and histopathological subtype) from the National Cancer Database [56], with higher diagnosed test accuracy than the nomogram developed in this study (Ovucevic—accuracy: 0.887 (95% CI: 0.880–0.893), Davey—0.840, 95% CI: 0.764–0.899). The same authors then updated their nomogram based on the TAILORx clinical cut-offs with slightly lower accuracy (0.860) [57]. Nevertheless, we must reiterate the importance of this nomogram for using the most crucial clinicopathological parameters (i.e., ER, PgR, menopause status, etc.) required to guide therapeutic decision-making in accordance with best practice management for those being treated for ER+/HER2−/N- disease in settings where routine RS testing is not available. 

In our multivariable analysis, a combination of menopause status, tumor grade, ER score, and PgR score independently predicted RS (accuracy: 0.800, 95% CI: 0.719–0.866) and RS > 25, respectively (accuracy: 0.858, 95% CI: 0.764–0.889). These results have been consistently observed in the previous studies which have built nomograms predictive of RS [42,50,51,56,57] and are unsurprising, as gene expression levels of ER and PgR were included in the algorithm designed by Paik et al. when creating the 21-gene expression assay [1]. Moreover, menopause status independently predicted RS, an unanticipated result which is less well-described in previous studies [22,24]. This is an interesting finding, as this data coincides with the recent results of the RxPONDER trial reported by Kalinsky et al. [58]. In RxPONDER, the authors describe an improved relative DFS benefit for premenopausal patients with RS < 25 in receipt of combined chemoendocrine therapy (vs. endocrine therapy alone) (HR: 0.54, 95% CI: 0.38–0.76), in addition to an early indication of an improved OS (HR: 0.47, 95% CI: 0.24–0.94) for those with 1–3 positive lymph nodes [3,58]. Nevertheless, caution is required when interpreting these results, as combined chemoendocrine therapy use in premenopausal patients in RxPONDER conferred an absolute risk reduction of just 5.2% and 1.3% for DFS and OS, respectively, in addition to a negligible benefit in those who were postmenopausal at diagnosis. Conversely, Carr et al. successfully challenged this concept in their analysis of 575 patients (142 premenopausal, 433 postmenopausal) which coherently demonstrates that menopause status is not useful in predicting RS in their analysis of 575 patients (142 premenopausal, 433 postmenopausal) [59]. Overall, data interpretation in relation to menopause status is challenging, given that confounding factors may change over time, with menses becoming inconsistent and unpredictable in those who are perimenopausal [60]. These challenges are reflected in the varying definitions and criteria used by several breast cancer study groups (including the National Comprehensive Cancer Network, Austrian Breast and Colorectal Cancer Study Group, and National Cancer Institute) to identify menopause [60]. This adds further complexity to interpretating the results and drawing of conclusions with respect to menopause status as a predictor of RS. Therefore, the authors recommend judicious use of menopause status as a parameter to determine chemotherapy benefit in the setting of early-stage ER+/HER2− disease, and advocate for endocrine and ovarian suppression therapy prescription as a useful alternative in these patients [61]. 

While the current study successfully developed a nomogram capable of predicting RS with high accuracy (0.840), sensitivity (0.858), and positive predictive value (0.971), there have been several previous attempts to develop surrogate biomarkers of RS. Klein et al. previously developed Magee equations from 817 patients with RS which were then successfully applied to differentiate low and high RS categories [22]. Similarly, Abubakar et al. developed an IHC4 score from four readily available IHC parameters (ER, PgR, HER2, and Ki-67) from 2498 patients with RS results through the substratification of patients into luminal-A-like (ER+/PgR+/HER2−/low-Ki-67) and luminal-B-like (ER+/PgR+ or PgR−/HER2+ or high-Ki-67) [62]. Interestingly, there is now evidence illustrating the evolving role of radiogenomic data which may be used to successfully predict RS [63,64]: Radiogenomics is an evolving field which examines the relationship between radiological features undetectable to the human eye and the underlying genomic landscape of the disease [65]. It is worth noting that proposed nomograms, Magee equations and IHC4 scores are all models which possess relatively straightforward capacity for prospective evaluation because they are easily reproduced with no additional expenses to healthcare services. Conversely, radiogenomics is imperfect in its current form and will inevitably require refinement of current techniques. This is likely to only be achieved through large-scale buy-in from international investment companies to financially facilitate the robust technological enhancement required (i.e.,: refining machine learning strategies through high-throughput image-based screening) to successfully challenge clinically validated multigene expression assays. However, if given the opportunity to be prospectively validated, it is plausible that radiogenomics has potential to become embedded into multidisciplinary discussion within clinical oncological practice to further facilitate a more personalized approach to oncological patient care. In the interim, RS testing will remain the gold standard, with use of surrogate biomarkers, such that the current nomogram may guide chemoendocrine prescription in the setting of early-stage ER+/HER2− disease.

Despite several strengths, the current analysis is subject to several limitations. Firstly, although RS was initially validated for use in patients being treated for ER+/HER2− breast cancer with axillary lymph node-negative disease, the paradigm has evolved in recent years to highlight the utility of the RS in cases of 1–3 positive lymph nodes in the axilla [58]. This study is retrospective in design, wherein all patients were recruited to this study in an era prior to validation of RS for use in cases of node-positive disease. Thus, the application of the nomogram developed in this study should ideally be limited to those with node-negative breast cancers. Secondly, this model has been developed and validated in a combined cohort of less than 450 patients all treated in a tertiary referral center serving a unique population on the edge of Europe [66,67]. Therefore, results generated from the genomic profile of these patients may be somewhat untranslatable to other culturally distinct regions, such as those in the United States and mainland Europe, limiting the clinical utility of this nomogram in certain jurisdictions. Thirdly, despite high diagnostic test accuracy, sensitivity, and PPV, this nomogram suffers from poor-to-modest specificity and NPV. It is possible that these may expose patients to overtreatment through the overestimation of high RS and failure to detect those with low-risk RS. Thus, prudent application of this nomogram is required. Finally, due to this nomogram being designed from a study comprised of retrospective data, this study inherently faces exposure to selection, ascertainment, and confounding biases. Nevertheless, the authors advocate for the application of this nomogram as a cost-effective surrogate to RS testing for use in poorly resourced healthcare economies to guide the personalisation of therapeutic decision-making in early-stage ER+/HER2− disease. 

In conclusion, this study successfully designed and validated a static and online user-friendly nomogram from routinely available clinicopathological parameters that could predict outcomes of the 21-gene RS expression assay with high diagnostic test accuracy, sensitivity, and PPV. Following the results of this study, a dynamic nomogram has been made readily available online, which may prove advantageous in serving patients being treated for early-stage ER+/HER2− disease in inadequately resourced and underfunded healthcare systems worldwide. Therefore, this study ensures the provision of a personalized approach to breast cancer patient care and attempts to address healthcare inequity in breast cancer management in a cost-effective, user-friendly manner. 

## Figures and Tables

**Table 1:** Characteristics of the entire cohort with matched training and test sets.

	Overall (*n* = 448)	Train Set (*n* = 315)	Test Set (*n* = 133)
Age at Diagnosis	58 (51, 64)	59 (51, 64)	58 (51, 65)
Menopause Status			
Premenopausal (0)	137 (31%)	91 (29%)	46 (35%)
Postmenopausal (1)	311 (69%)	224 (71%)	87 (65%)
Diagnostic pathway			
Symptomatic (0)	289 (65%)	208 (66%)	81 (61%)
Screening Detected (1)	159 (35%)	107 (34%)	52 (39%)
Main Tumor Size	20 (14, 26)	20 (15, 26)	19 (13, 26)
Tumor Stage			
T1	219 (49%)	148 (47%)	71 (53%)
T2	218 (49%)	159 (50%)	59 (44%)
T3	11 (2.5%)	8 (2.5%)	3 (2.3%)
Histological Subtype			
IDC	341 (76%)	243 (77%)	98 (74%)
ILC	82 (18%)	52 (17%)	30 (23%)
Other	25 (5.6%)	20 (6.3%)	5 (3.8%)
Tumor Grade			
Grade 1	44 (10%)	28 (9.5%)	16 (13%)
Grade 2	296 (70%)	206 (70%)	90 (71%)
Grade 3	82 (19%)	62 (21%)	20 (16%)
ER Score			
2	2 (0.4%)	1 (0.3%)	1 (0.8%)
3	2 (0.4%)	1 (0.3%)	1 (0.8%)
4	2 (0.4%)	2 (0.6%)	0 (0%)
5	3 (0.7%)	1 (0.3%)	2 (1.5%)
6	16 (3.6%)	12 (3.8%)	4 (3.0%)
7	56 (12%)	43 (14%)	13 (9.8%)
8	367 (82%)	255 (81%)	112 (84%)
PgR Score			
0	62 (14%)	46 (15%)	16 (12%)
2	6 (1.3%)	3 (1.0%)	3 (2.3%)
3	21 (4.7%)	12 (3.8%)	9 (6.8%)
4	26 (5.8%)	19 (6.1%)	7 (5.3%)
5	48 (11%)	36 (11%)	12 (9.1%)
6	62 (14%)	36 (11%)	26 (20%)
7	73 (16%)	55 (18%)	18 (14%)
8	148 (33%)	107 (34%)	41 (31%)
OncotypeDX© Recurrence Score	17 (13, 22)	17 (13, 22)	17 (13, 22)

Legend: N; number, median (IQR); interquartile range, IDC; invasive ductal carcinoma, ILC; invasive lobular carcinoma, T; tumor stage, ER; estrogen receptor, PgR; progesterone receptor.

**Table 2:** Multivariable linear regression analysis to predict clinicopathological data capable of predicting continuous Recurrence Score.

Surrogate RS Model	Beta	95% CI _1_	*p*-Value
Age at Diagnosis	−0.01	−0.02, 0.00	0.12
Menopause Status			
Postmenopausal (0)	-	-	-
Premenopausal (1)	0.25	0.03, 0.48	0.028
Symptomatic Presentation			
Screening Detected (0)	-	-	-
Symptomatic (1)	0.09	−0.06, 0.23	0.2
Tumor Stage			
T1	-	-	-
T2	0.03	−0.10, 0.15	0.7
T3	0.18	−0.23, 0.59	0.4
Tumor Grade			
Grade 1	-	-	-
Grade 2	0.09	−0.13, 0.30	0.4
Grade 3	0.28	0.03, 0.52	0.026
Histological Subtype	0.99	0.50, 1.72	0.9
Increasing ER Score	−0.14	−0.22, −0.06	0.001
Increasing PgR Score	−0.02	−0.04, 0.00	0.11

Legend: _1_ CI = confidence interval, RS; recurrence score, ER; estrogen receptor, PgR; progesterone receptor.

**Table 3:** Multivariable logistic regression analysis to predict clinicopathological data capable of predicting recurrence score > 25.

Surrogate RS > 25 Model	OR _1_	95% CI _1_	*p*-Value
Age at Diagnosis	1	0.95, 1.05	>0.9
Menopause Status			
Postmenopausal (0)	-	-	-
Premenopausal (1)	1.4	0.40, 5.23	0.6
Symptomatic Presentation			
Screening Detected (0)	-	-	-
Symptomatic (1)	0.87	0.40, 1.96	0.7
Tumor Stage			
T1	-	-	-
T2	1.37	0.47, 3.99	0.6
T3	3.99	0.07, 164	0.5
Tumor Grade			
Grade 1	-	-	-
Grade 2	2.22	0.57, 14.8	0.3
Grade 3	5.67	1.32, 40.0	0.037
Histological Subtype	0.98	0.51, 1.76	>0.9
Decreasing ER Score	1.33	1.02, 1.66	0.05
Decreasing PgR Score	1.16	1.06, 1.25	0.002

Legend: _1_ OR = odds ratio, CI = confidence interval, RS; recurrence score, ER; estrogen receptor, PgR; progesterone receptor.

## Data Availability

Data made available upon reasonable request to the corresponding author.
